# Unlocking the Potential of Carbon Quantum Dots for Cell Imaging, Intracellular Localization, and Gene Expression Control in *Arabidopsis thaliana* (L.) Heynh.

**DOI:** 10.3390/ijms242115700

**Published:** 2023-10-28

**Authors:** Zhimin Lin, Muhammad Moaaz Ali, Xiaoyan Yi, Lijuan Zhang, Shaojuan Wang, Faxing Chen

**Affiliations:** 1 Fujian Academy of Agricultural Sciences Biotechnology Institute, Fuzhou 350003, China; 2 College of Horticulture, Fujian Agriculture and Forestry University, Fuzhou 350002, China; muhammadmoaazali@yahoo.com (M.M.A.); 1210305019@fafu.edu.cn (X.Y.); 1210305020@fafu.edu.cn (L.Z.); 3210330057@fafu.edu.cn (S.W.); cfaxing@126.com (F.C.)

**Keywords:** carbon dots, 4′,6-diamidino-2-phenylindole (DAPI), cell localization, transcriptome analysis, argonaute family, DNA methylation, transmission electron microscopy

## Abstract

Utilizing carbon quantum dots (CQDs) as biomaterials for delivering small substances has gained significant attention in recent research. However, the interactions and mechanisms of action of CQDs on plants have received relatively little focus. Herein, we investigated the transportation of CQDs into various organs of *Arabidopsis thaliana* (L.) Heynh. via the vessel system, leading to the epigenetic inheritance of Argonaute family genes. Our findings reveal that CQDs may interact with microRNAs (miRNAs), leading to the repression of post-transcriptional regulation of target genes in the cytoplasm. Transcriptome and quantitative PCR analyses demonstrated consistent gene expression levels in offspring. Moreover, microscopic observations illustrated rapid CQD localization on cell membranes and nuclei, with increased nuclear entry at higher concentrations. Notably, our study identified an alternative regulatory microRNA, microRNA172D, for the Argonaute family genes through methylation analysis, shedding light on the connection between CQDs and microRNAs.

## 1. Introduction

Nanotechnology and nanomaterials have seen extensive utilization in recent years [1,2]. Ever since the breakthrough discovery of carbon quantum dots in 2014, they have found applications across various domains, including biomedicine, sensor technology, optics, and catalysis [3,4]; the ability to penetrate cells; and rapid distribution in organisms [5]. Carbon-based quantum dots (CQDs) are generally classified as bright fluorescent carbon dots (FCDs) and graphene quantum dots (GQDs) [6,7]. CQDs are characterized by low toxicity, low cost, and chemical inertness, especially biocompatibility. So far, nanotechnology research has mainly been used to provide new methods for using nanoparticles as tools for gene delivery [8] and their applications in cell and animal biology [9]. Lately, CQDs have garnered attention for their potential role as regulators of plant growth in agriculture [10,11,12,13]. Nevertheless, there remains a scarcity of research investigating the interactions and mechanisms through which nanomaterials affect plants [14]. The remarkable attributes of CQDs offer a fresh perspective on the connections between plants and nanomaterials. In this context, transcriptome profiling emerges as a crucial method for comprehending the functional components of the genome, shedding light on alterations in the expression levels of various transcripts, including mRNAs, non-coding RNAs, and small RNAs, as detailed in reference [15].

*Arabidopsis thaliana* (L.) Heynh., a compact cruciferous plant, serves as an excellent choice for conducting research in the realms of plant molecular genetics, development, physiology, and biochemistry [16,17,18]. In the context of plant research, it is essential to understand the various RNA interference (RNAi) silencing pathways [19]. These pathways include cytoplasmic siRNA silencing [20], endogenous messenger RNAs by miRNAs [21], and DNA (deoxyribonucleic acid) methylation and transcriptional repression [22]. When mature small RNAs (smRNAs) are incorporated into the RNA-induced silencing complex (RISC), it participates in the regulation of the target gene expression at transcriptional or post-transcriptional levels [23].

Argonaute (AGO) proteins have been identified in plants, animals, and fungi [24,25]. Studies have consistently demonstrated that AGO proteins, acting as a central component of RISCs, play a pivotal role in mediating the RNA interference (RNAi) process [26], and are associated with three pathways of RNA silencing [19]. Arabidopsis contains 10 AGO proteins but mainly encodes 9 functional AGO proteins [27]. Arabidopsis AGOs are divided into three clades: AGO1, AGO5, and AGO10 are classified in the first clade; AGO2, AGO3, and AGO7 are classified in the second clade; AGO4, AGO6, AGO8, and AGO9 are classified in the third clade [28]. *LBD21-1*, as a transcription factor, promotes the transcriptional expression level of AGO10 protein by binding to the promoter region of AGO10 under salt stress conditions [29]. *HOS1* acts as a regulator of miRNA, and it can regulate the *AGO1* expression levels by affecting the transcription of *MIR168b* [30]. The 4′,6-diamidino-2-phenylindole (DAPI) was first synthesized in 1971 and binds to AT-rich DNA [31], and it has been used as a fluorochrome for mitochondrial or nuclear DNA [32].

The investigation into the intracellular localization and tracking of CQDs within plant cells and their impact on plant gene expression mechanisms is relatively scarce. The objectives of this study were to investigate the transport of CQDs within *Arabidopsis thaliana*, their influence on the inheritance of Argonaute family genes, and their potential role in microRNA-mediated gene regulation. Additionally, the study aimed to assess the stability of gene expression in the offspring; observe the cellular localization of CQDs, particularly under high concentrations; and identify a regulatory microRNA, microRNA172D, through methylation analysis. This research not only offers a theoretical foundation for potential applications of carbon nanomaterials in plants, but also lays the groundwork for enhancing the precise targeting of carbon nanomaterials within plant systems in the future.

## 2. Results

### 2.1. Characterization of CQDs

**Figure 1:**
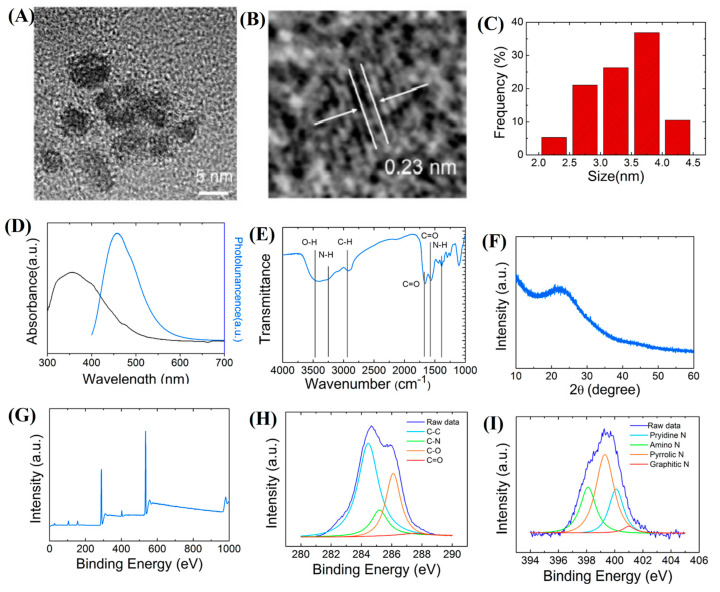
Characterization of CQDs: (**A**) TEM and (**B**) HRTEM (inset) images. Scale bar = 5 nm. The arrows indicate a crystal lattice size of 0.23 nm. (**C**) The corresponding size distribution. (**D**)UV−VIS absorption and PL spectra. (**E**) FTIR spectra. (**F**) XRD patterns. (**G**) XPS spectra. (**H**) C 1s peaks signal. (**I**) N 1s peaks signal from the XPS analysis.

According to the transmission electron microscopy (TEM) (Figure 1A,B) and its corresponding size distribution image (Figure 1C), the CQDs synthesized were at a homogeneous and narrow distribution with an average size of 3.5 nm. The lattice spacing (marked by white arrows) corresponded to (100) facets of CQDs, demonstrating the crystallinity and formation of the materials, as depicted in Figure 1B [33,34]. The UV–VIS absorption spectra of CQDs (in Figure 1C) illustrate a distinct excitonic absorption band centered at 351 nm, indicating the high conjugation and quality of CQDs [35]. The photoluminescence (PL) emission of the CQD peak with an emissive peak was centered at about 458 nm with an excitation wavelength of 405 nm (Figure 1D). The Fourier transform infrared (FT-IR) spectra are shown in Figure 1E. The absorption peaks located at 3410 and 3280 cm^−1^ were assigned to the strong stretching vibration bands of O-H and N-H, respectively. The observed strong bands at 1560, 1380, and 1659 cm^−1^ were ascribed to C=N, C-N, and -COO- stretching vibrations, respectively [36,37]. The X-ray powder diffraction (XRD) pattern of the CQDs exhibited a broad (002) characteristic peak centered at around 24° (Figure 1F). The X-ray photoelectron spectroscopy (XPS) surveys confirmed the FT-IR data further (Figure 1G), in which the three prominent peaks were attributed to C 1s, O 1s, and N 1s, demonstrating that the primary compositions of CQDs were C, O, and N. The C 1s XPS spectrum in Figure 1H was deconvoluted into four peaks at 288.1, 286.6, 285.3, and 284.2 eV, which represent the C 1s states in C=O, C-O, C-N, and C-C, respectively [38]. The four deconvoluted peaks in Figure 1I belong to the N 1s states in pyridine N, amino N, pyridine N, and graphite N structure [39]. Many functional groups, such as -COOH, -OH, and -NH_2_-passivating the surface of the CQDs, as certified by the FTIR along with XPS, indicated meaningful application in hydrogen bonding with biomolecules.

### 2.2. Cell Localization of CQDs in Arabidopsis thaliana

**Figure 2:**
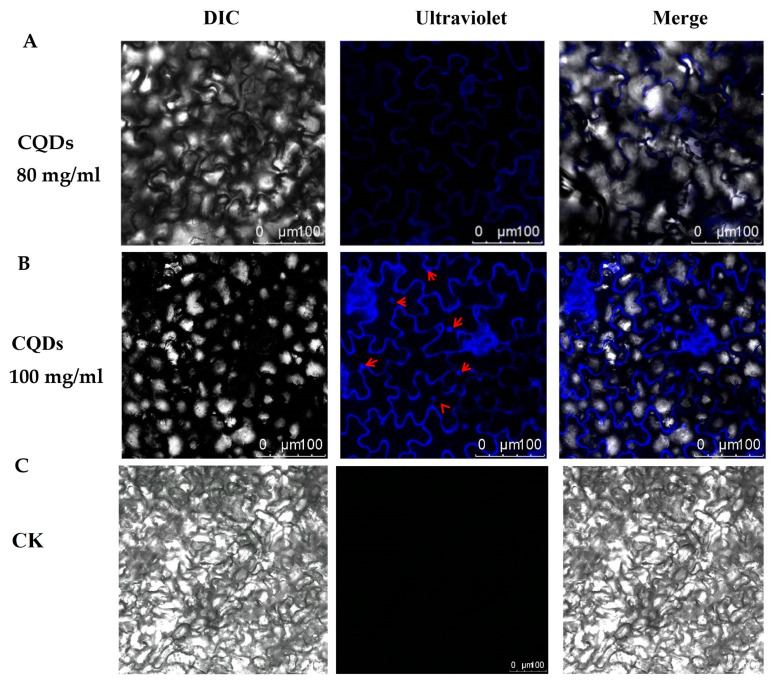
Cell localization of CQDs in *Arabidopsis*. (**A**) CQDs at a concentration of 80 mg/mL are localized to the cell membrane. (**B**) CQDs at a concentration of 100 mg/mL are localized to the cell membrane and nucleus. (**C**) Morphology of CK (control) cells injected without CQDs. Scale bar—100 μm. The red arrow represents the nucleus. The excitation wavelength of ultraviolet is 405 nm. DIC stands for visible light.

We investigated the intracellular distribution of CQDs by employing leaf injection as our experimental method. The findings indicated that the distribution pattern of CQDs within cellular organelles exhibited a concentration-dependent relationship. Specifically, at lower concentrations, such as 80 mg/mL, CQDs predominantly accumulated within the cell membrane (Figure 2A). However, at high concentrations, i.e., 100 mg/mL, CQDs appeared significantly enriched in the nucleus in addition to the cell membrane (Figure 2B). The results of the repeated experiments are provided in Figure S1. Water-soluble CQDs have been shown to move freely through the organelles of *Arabidopsis* due to their small nanoparticle size.

### 2.3. Co-Localization Analyzation of AGO3/GFP Fusion Proteins and CQDs

**Figure 3:**
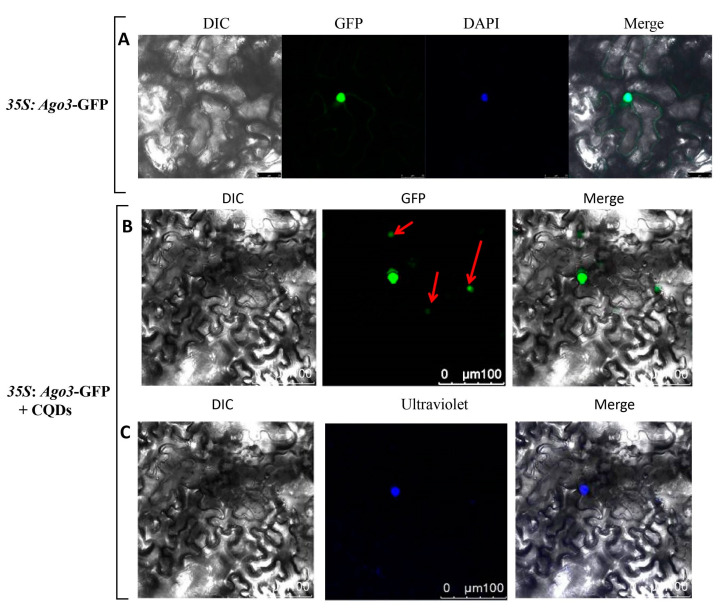
Localization of the AGO3-GFP fusion protein and CQDs in *N. benthamiana* cells. (**A**) The *AtAGO3* gene, when fused to green fluorescent protein (GFP) and under the control of the 35S promoter, displays nuclear localization, with DAPI serving as an internal reference control. (**B**) The *AtAGO3* gene, when fused with GFP and regulated by the 35S promoter, exhibits nuclear localization. (**C**) The expression of the *AtAGO3* gene, under the control of the 35S promoter and fused with GFP, is determined through the simultaneous injection of carbon quantum dots (CQDs). This reveals nuclear localization under two distinct excitation wavelengths: 488 nm (for GFP) and 405 nm (for CQDs). Scale bar, 100 µm. The red arrows and blue dots indicate the presence of GFP and CQDs in the nucleus, respectively.

To further verify the localization of CQDs in plant cells, we used *Nicotiana benthamiana* as a test material. Using DAPI as an internal reference, the results indicate that *AtAGO3* was a nuclear-localized gene (Figure 3A). Co-localization of the AtAGO3 protein with CQDs showed that they tended to cluster in the nucleus. The experimental results show that the CQDs were negatively charged, whereas the GFP proteins were positively charged. Therefore, under the influence of an electric field, CQDs tended to accumulate around the nucleus (Figure 3B,C). Thus, further experiments showed that the range of motion of CQDs was closely related to their concentration.

### 2.4. The Moving Trail of CQDs in Arabidopsis thaliana

**Figure 4:**
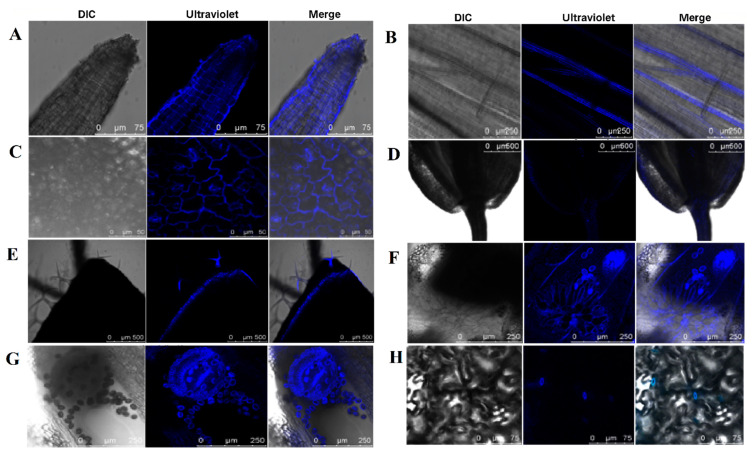
Distribution of CQDs injections in *Arabidopsis thaliana* after 3 days. (**A**) Microscopic observation of CQDs on apical tissue. (**B**) Microscopic observation of CQDs on the stem. (**C**) Microscopic observation of CQDs on leaf. (**D**) Microscopic observation of CQDs on the flower stalk tissue. (**E**) Microscopic observation of CQDs on the leaf epidermal hairs. (**F**) Microscopic observation of CQDs on the flower tissue. (**G**) Microscopic observation of CQDs on anther tissue. (**H**) Microscopic observation of CQDs on the stomata tissue. Here, 405 nm is used for excitation light in ultraviolet, and DIC stands for visible light. The scale bars for panels A, B, C, D, E, F, G, and H are 75, 250, 50, 500, 500, 250, 250, and 75 µm, respectively.

The entry of CQDs into Arabidopsis plants was demonstrated in two different ways. It is shown that CQDs were taken up by Arabidopsis roots via transmembrane transport, mainly from the root tip margin or root cell-by-cell delivery pathway (Figure 4A). When injected from the leaves, the CQDs were mainly transported by the phloem (Figure 4B). Confocal observations revealed that CQDs could be transported into different organs of Arabidopsis, including the Apical (Figure 4C), stem (Figure 4D), leaf (Figure 4E), flower stalk (Figure 4F), leaf pubescence (Figure 4G), and stomata (Figure 4H). The fluorescence of pollen grains shows that CQDs can entered not only anther tissue, but also pollen grains, providing epistasis for their influence on gene expression in Arabidopsis.

### 2.5. Transcriptome Analysis

Heatmap analysis of the transcriptomic data was carried out for the two experimental groups, i.e., CQDs and CK (control) (Figure S2A). Among all of the differentially expressed genes, we focused on the Argonaute family gene, *AtAGO3*, which was significantly differentially expressed between the two groups (Figure S2B).

The classification from the GO (Gene Ontology) enrichment showed that the main differentially expressed genes (DEGs) were clustered into three major categories: biological processes, cellular components, and molecular functions (Figure S2C), including cells, receptors, enzymes, etc. The results suggest that CQDs could stimulate changes in the transcription level of genes at a cellular or molecular level. The top 44 enriched KEGG (Kyoto Encyclopedia of Genes and Genomes) pathways associated with DEGs showed that the effects were focused on environmental information processing, cellular processes, genetic information processing, metabolism, and organismal systems. The “Environmental information processing” contained the largest numbers of DEGs comparatively different between CQDs and CK (Figure S2D).

### 2.6. Gene Expression Validation through RT-qPCR

**Figure 5:**
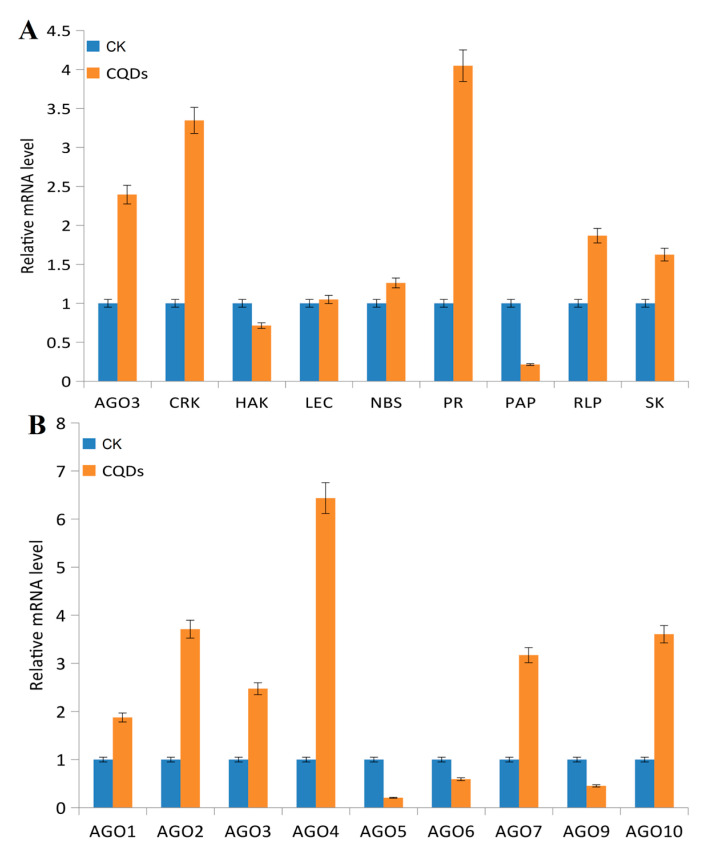
RT-qPCR analysis of 9 different significantly affected genes (**A**) as well as 9 Argonaute family genes (**B**) of *Arabidopsis* under the influence of CQDs. The *Arabidopsis* actin gene is used as an internal reference and three replicates of each sample are used. Abbreviations: AGO3—Argonaute 3; CRK—Cysteine-rich receptor-like protein kinase; HAK—potassium transporter; LEC—leucine-rich repeat disease resistance protein-like; NBS—TIR-NBS class of disease resistance protein; PR—uncharacterized protein; PAP—purple acid phosphatase; RLP—receptor-like protein; SK—putative WRKY transcription factor.

To validate the expression profiling results, we selected nine genes of *Arabidopsis thaliana* that were significantly up- and down-regulated between CK and CQDs groups for further analysis (Figure 5A). The results of RT-qPCR confirmed that the expressions of the aforementioned genes, including *AtAGO3*, were altered. As most of the genes in the Argonaute family were expressed in the nucleus and belonged to the functional organelles of the cell, this was an advantage for our subsequent studies. One of the up-regulated genes from the Argonaute family was further selected as a target to analyze the mechanism of CQD influence.

In *Arabidopsis thaliana*, the Argonaute family gene consisted of a total of nine genes. The RT-qPCR analysis results showed that *AtAGO1*, *AtAGO2*, *AtAGO3*, *AtAGO4*, *AtAGO7*, and *AtAGO10* were up-regulated, and *AtAGO5*, *AtAGO6,* and *AtAGO9* were down-regulated by CQDs (Figure 5B). This suggests they were affected differently by CQDs in *Arabidopsis*.

### 2.7. Analysis of Genetic Properties of Targeted Genes

**Figure 6:**
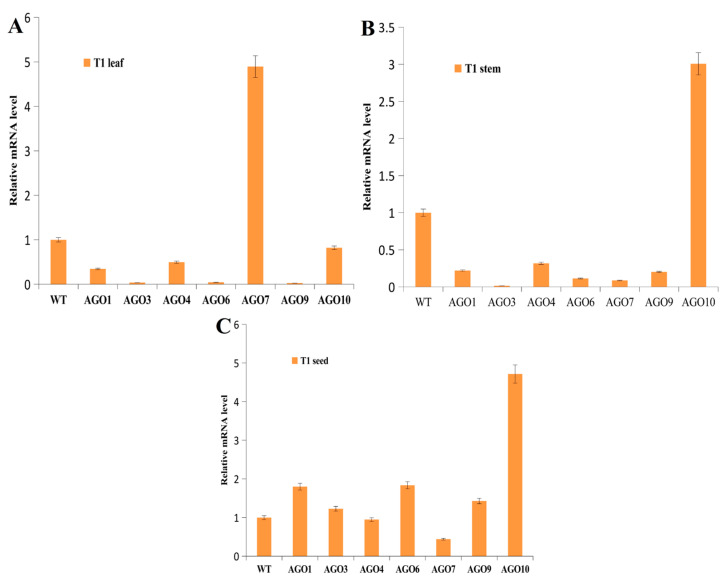
RT-qPCR expressions of Argonaute family genes in the leaves (**A**), stems (**B**), and seeds (**C**) of *Arabidopsis thaliana*. The *Arabidopsis* actin gene was used as an internal reference and three replicates of each sample were used.

Quantitative expression analysis revealed that in the second generation (T1) of *Arabidopsis thaliana*, seven genes within the Argonaute family, namely *AtAGO1*, *AtAGO3*, *AtAGO4*, *AtAGO6*, *AtAGO7*, *AtAGO9*, and *AtAGO10*, exhibited consistent gene expression levels when compared to the control (CK). In the leaves, except for *AtAGO7*, the other six genes were down-regulated compared with the control (Figure 6A). In the stems, except for *AtAGO10*, the other six genes were down-regulated compared with the control (Figure 6B). However, the expression patterns in the seeds were quite specific, the *AtAGO7* gene was down-regulated and the other six genes were up-regulated (Figure 6C). Interestingly, *AtAGO2* and *AtAGO5* had no detectable expression in the leaves, which may be related to their tissue-specific expression. All of this suggests that the effects of CQDs on the genes of *Arabidopsis* will continue to occur in the next generation as well.

### 2.8. DNA Methylation

**Figure 7:**
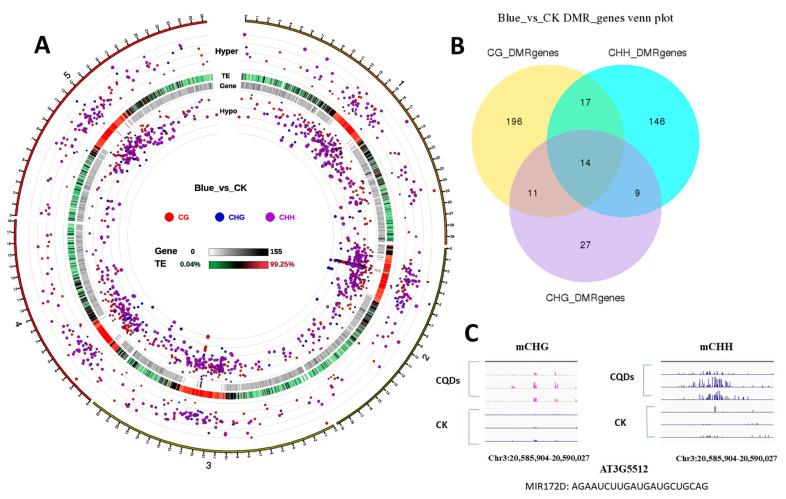
DNA methylation analysis between CQDs and CK. (**A**) Circos plot of gene density; gene and TE density; and methylation levels of CG, CHG, and CHH between CQDs (blue) and CK. (**B**) Venn diagrams showing the overlap among differential expression genes. (**C**) Screenshot of DNA methylation levels over one representative gene, *AT3G5512*. The red and blue bars indicate TEs and genes, respectively.

The methylation analysis revealed a large number of gene methylations between CQDs and CK, including CG, CHG, and CHH (Figure 7A,B). We detected microRNA172D using differential genes between CQDs and CK through DNA methylation analysis (Figure 7C). The Venn diagram shows that the methylation distribution of CQDs was found on CG, CHH, and CHG compared with CK, but there were relatively few methylated genes in CHG. Through gene analysis, we selected our nine genes of interest distributed between CHH and CHG in microRNA172D. The methylation analysis showed that mCHG and mCHH in the region between 20,585,904-20,590,027 on chromosome 3 were significantly increased in methylation between CQDs and CK.

### 2.9. Transient Overexpression of microRNA172D in Arabidopsis

**Figure 8:**
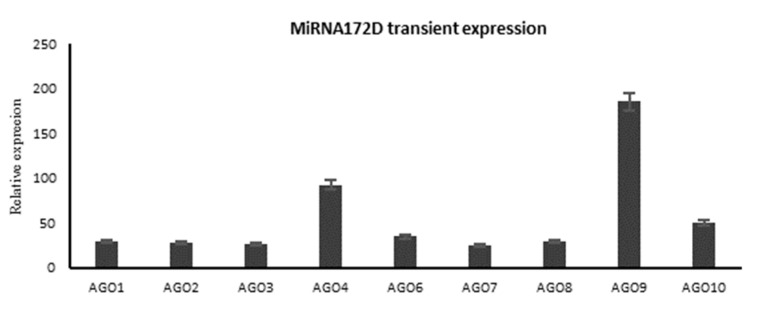
The microRNA172D transient expression of Argonaute family genes in *Arabidopsis* leaves.

To further verify the true relationship between microRNA172D and Argonaute genes, we cloned the precursor of microRNA172D and *A*. *tumefaciens*-mediated transient expression of microRNA172D driven by the 35S promoter injected into *Arabidopsis* leaves. The results of RT-qPCR verified that the transient overexpression of microRNA172D induced a response in the Argonaute family genes, with the exception of *AtAGO5* (Figure 8).

### 2.10. Hypothetical Model of CQDs in Plant Bodies and Cells

**Figure 9:**
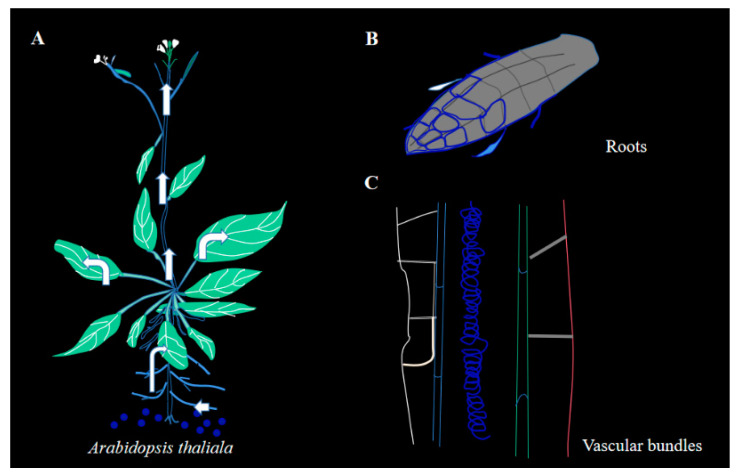
Transport of CQDs in *Arabidopsis thaliana*. (**A**) Transport of CQDs to different organs in Arabidopsis. (**B**) Transport of CQDs in the root system of *Arabidopsis thaliana*. (**C**) CQDs are transported by vascular bundles in *Arabidopsis* stems.

**Figure 10:**
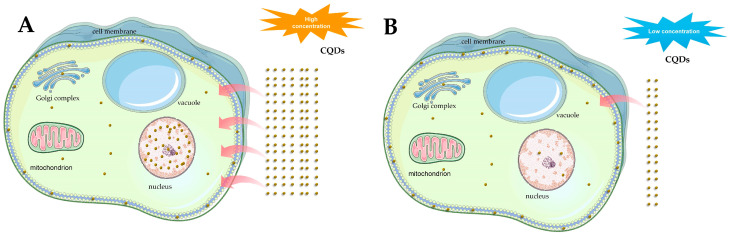
Differential localization of CQDs in cells after injection of low and high amounts of CQDs. (**A**) They mainly enter the cell membrane and nucleus under high concentrations of CQDs. (**B**)When CQDs are at low concentrations, they mainly accumulate in the cell membrane.

CQDs itself is hydrophilic and negatively charged, and the characteristics of plant cells create favorable conditions for CQDs (Figure 9A). So, this also allows CQDs to quickly reach every organ in the plant. Once CQDs enter the plant, they are transported by two main routes i.e., from the roots and vascular bundles. The CQDs are then localized in organelles such as the cell membranes or nucleus, depending on the concentration of CQDs (Figure 9B,C). Meanwhile, the results show that when the CQDs were in a low concentration (80 mg/mL), they were mainly located on the cell membrane (Figure 10A), then partially moved into the cells, including the nucleus, but they could not be observed by confocal observation. When the CQDs were at a high concentration (100 mg/mL), they were mainly located in the cell membrane and nucleus (Figure 10B).

## 3. Discussion

In the context of this research, the CQDs were synthesized with an average size of 3.5 nm. To render these CQDs water soluble, multiple hydrophobic groups were introduced [40,41,42,43]. The design helped the CQDs to easily enter the plant cell due to their hydrophilic property. In addition, the blue emission of CQDs was convenient for observation when multiple fluorescent emissions were used in a single sample.

The mechanism of transport through the cell membrane is usually divided into two categories: passive transport and active transport. Non-polar molecules penetrate more easily than polar ones, and small molecules without polarity can pass through a lipid bilayer, but it is impermeable to charged materials such as ions [44]. CQDs bind to the surface of the cell’s outer membrane due to their hydrophilic and negatively charged properties. CQDs pass through the cell membrane into the cell using their hydrophilicity, and once inside the cell, the two properties of hydrophilicity and charge play a role in guiding the CQDs to penetrate the cytoplasm/nucleus. Currently, most nanomaterials, especially CQDs, are mainly used as gene-delivery tools [45]. In this study, we used *Arabidopsis thaliana* and *Nicotiana benthamiana* as plant materials. After injecting the CQD-water solution into their leaves, the results revealed the presence of CQDs within plant cells and provided insights into how they affect plant genes. The main finding lies in the predominant transport pathways of CQDs within cells, primarily involving two routes: apoplast and transmembrane transport. When CQDs were introduced into the plant leaves, they quickly distributed themselves within the cell membrane and nucleus in a matter of minutes. In addition, the high concentration could obviously improve the ability of CQDs to localize in the nucleus. As the concentration increased, the amount that moved into the nucleus was more obvious. In addition, transcriptome analysis revealed its regulatory impact on genes in Arabidopsis responsible for encoding proteins localized in the nucleus and membrane, with a particular focus on stress- and disease-resistance-related genes (Figure 5A).

Importantly, the influence of CQDs on the expression of plant genes is heritable. Quantitative analysis of the AGO family genes in T1 leaves, stems, and seeds showed that most of these genes continued to be regulated at different expression levels, especially in seeds (Figure 5B). The methylation sequencing results showed that CQDs entering plant cells directly caused methylation of the plant genes, including small RNAs, e.g., microRNA172D was significantly methylated compared with the control (Figure 7C). Not many studies have shown that CQDs cause gene methylation in plants. The only research is on how to modify the CQDs to detect the methylation level of genes [46].

DAPI-DNA complexes emit maximum blue fluorescence at around 450 nm [47]. CQDs have the same blue emission characteristics as DAPI at 405 nm but are non-toxic to plant cells compared with DAPI. Therefore, CQDs or their improvements can be developed to replace DAPI in plant cell co-localization. In addition, unlike the widely used Q-point, the C-point itself contains no heavy metal components, so it can remain low or non-toxic to plant or animal tissues and organs [48]. Some metals that are toxic to plants, such as arsenic, can be effectively reduced by the combined use of point C and As for bio-toxic applications of arsenic, thus promoting plant growth [49]. In addition, amino CQDs synthesized from amino and carboxyl groups have shown stable fluorescence parameters in both dilute alkali metal solutions and seawater [50]. The unique properties of quantum dots, nanocrystals with high brightness and transformable fluorescence properties, have led to their widespread use in plant research, biomedicine, and industry, and thus research into the mechanism of action of quantum dots in cells, tissues, and organisms is essential [51]. In conclusion, although many nanomaterials inhibit plant growth, disrupt plant cell structure, and cause damage to plants, they have been widely used in various fields and in-depth research into their mechanisms is important for the environment and human health [52].

## 4. Materials and Methods

### 4.1. Experiment Material, Instruments, and Chemical Reagents

The CQDs (CAS:7440-44-0) were obtained from Jiangsu Xianfeng Nanomaterials Technology Co., Ltd. (Nanjing, China) *Arabidopsis* Columbia-0 (Col-0) ecotype and *Nicotiana benthamiana* were cultured in a greenhouse under long-day (16/8 h) conditions. These were grown at about 25–26 °C for four weeks. The optimum light intensity in the growth chamber was approximately 120–150 μmol/m^2^/s.

The morphology characterization of CQDs was achieved using a transmission electron microscope (Tecnai G2 F20). Photoluminescence (PL) spectra were recorded with a Hitachi F-4600 fluorescence spectrophotometer. The UV-VIS absorption spectra were tested with a UV/VIS/NIR spectrophotometer (Shimadzu, Columbia, MD, USA, UV-3600). X-ray diffraction (XRD) spectra were collected with an X’Pert PRO diffractometer (PANalytical). Fourier transform infrared spectroscopy (Nicolet is 50) was used to collect Fourier transform infrared (FT-IR) spectra by dropping CQDs on KBr transparent substrates in transmission mode [53].

CQDs (100 mg/mL, 80 mg/mL and 10 mg/mL), 1 μg liner DNA, 1 μg circular DNA, CQDs + 1 μg liner DNA, and CQDs + 1 μg circular DNA were prepared. All of these were electrophoresed in a 1.5% (*w*/*v*) agarose gel (Biowest, Riverside, MO, USA) at 100 V for 30 min, and photographed under UV conditions (Figure S3). TAE (Tris-acetate-EDTA) buffer containing 40 mM Tris, 20 mM acetic acid, and 1 mM EDTA was used for both the running buffer and agarose gel preparation.

CQDs were introduced into the plants using a standard syringe, benefiting from their water-soluble properties for easy integration with DNA. The DNA, selected randomly from conventional plasmids, was blended with CQDs to confirm that CQDs do not form bonds with DNA, but instead maintain their free dispersion within plant cells. It is worth noting that CQDs exhibit blue light emission at 405 nm, a distinctive feature absent in DNA. The objective was to underscore both the compatibility of CQDs with DNA and the unique optical properties of CQDs in comparison with DNA within the plant cell environment.

### 4.2. Transcriptome Analysis

The 3 days following the injection of CQDs into *Arabidopsis* leaves were used for cDNA library construction. Plants that were not subjected to injection served as the control (CK). Illumina 280 bp read pair technology was used to sequence the cDNA libraries. Low quality data with a qual value of less than 20 and consisting of short reads (length < 35 bp) were filtered from the raw data. Clean reads were aligned to the *Arabidopsis* genome using bowtie2 and tophat 2.0.14, htseq-count was then used to calculate the expected fragments per kilobase of mapped FPKM, and the edge R package in R was applied to identify DEGs with fold-change ≥2.0 or ≤2.0 (log_2_FC ≥ 1) and Benjamini-Hochberg corrected False Discovery Rate (FDR) ≤ 0.01. We used AgriGO (http://bioinfo.cau.edu.cn/agriGO/, accessed on 23 January 2023) and KOBAS 3.0 (http://kobas.cbi.pku.edu.cn, accessed on 23 January 2023) to perform GO functional enrichment analysis and KEGG enrichment analysis with default parameters.

### 4.3. Identification of DEGs

Clean reads were mapped to the unigenes library using Bowtie and the read count of each gene was derived by mapping the results using RSEM. Fragments per kilobase of transcript per million mapped reads (FPKMs) values were calculated for each of the unigenes to determine the expression profiles, and the differences in gene expression between CK and carbon treatments were analysed using DESeq using the FDR method. DEGs were identified using FDR < 0.01 and fold change (FC) ≥ 2 as thresholds, and cluster analysis was performed based on the differential expression of unigenes across samples. Unigenes expression was analysed in CK and CQDs treatment using Bowtie and RSEM software (version 0.6) and normalized to FPKM. Differential gene expression was assigned when (i) FDR values were <0.01 and (ii) fold-change (FC) was ≥2. The expression character of DEGs in *Arabidopsis thaliana* was defined as up-regulated or down-regulated according to their high or low levels, respectively [54]. DEGs expressed at higher levels in *Arabidopsis* were defined as up-regulated and DEGs expressed at lower levels in *Arabidopsis* were defined as down-regulated.

### 4.4. Subcellular Localization Analysis

The fluorescence emanating from the green fluorescent protein (GFP) and carbon quantum dots (CQDs) within transfected cells of either Arabidopsis or *Nicotiana benthamiana* was examined 48 h post-transfection, utilizing a Leica confocal microscope (TCSSP8, Leica Geosystems, Switzerland). DAPI, a fluorescent stain with a strong affinity for DNA, and CQDs were stimulated under ultraviolet (UV) illumination at a wavelength of 405 nm. Conversely, GFP was stimulated at 488 nm. For the purposes of fluorescence microscopy, DAPI was dissolved within a 0.025 M Tris-HCI buffer with a pH of 7.0. Subsequently, it was blended with other constituents on microscope slides to attain a final concentration of 50 pg/mL [55].

### 4.5. DNA Methylation

The DNeasy Plant Mini Kit (QIAGEN, Hilden, Germany) was used for DNA extraction. The genomic DNA was randomly interrupted at 200–300 bp using S220 Focused-ultrasonicator (Covaris, Brighton, UK); the interrupted DNA fragments were end-repaired, A-tailed, and ligated to sequencing junctions with all cytosines modified by methylation. After bisulfite treatment (using EZ DNA Methylation Gold Kit, Zymo Research, Beijing, China), the unmethylated C became U (T after PCR amplification), while the methylated C remained unchanged, and the final DNA library was amplified by PCR. Quantitative assays were then performed. Initial quantification was performed using Qubit 2.0 Fluorometer (Thermo Fisher Scientific, Shanghai, China), diluting the library to 1 ng/μL, followed by detection of the library insert length using a 2100 Bioanalyzer (Agilent, Santa Clara, CA, USA). Different libraries were pooled according to the effective concentration and the target downstream data volume required for Illumina HiSeq/MiSeq sequencing. For methylation site detection, many kinds of analyses were performed, including methylation site IGV (Integrative Genomics Viewer) visualization, methylation density circos plot, functional region heat map, differential methylation level circos plot, DMR (differentially methylated region) anchored region distribution, DMR-related gene GO enrichment, KEGG enrichment, etc. Methylation sequencing was performed by Novozymes Biotechnology Co., Ltd., Beijing, China.

### 4.6. Vector Construction and Transient Transformation

For miRNA overexpression, the precursor microRNA172D or MIR172D was amplified by PCR using primers miF2 and miR2. The PCR product was cloned into pCXUN containing the maize ubiquitin promoter to construct the overexpression vector by TA cloning (Figure S4). *Agrobacterium tumefaciens* ((Smith and Townsend) Conn.) G3101 was transformed and injected into tobacco. A single microRNA172D overexpressing colony was picked and incubated overnight at 250 rpm/min on LB medium with 50 μg/mL of kanamycin and 100 μg/mL of rifampicin, centrifuged to collect the organisms, lysed in infection medium (4.88 g MES, 2.5 g glucose, 0.2 g NaH_2_PO_4_), allowed to stand for 2 h at room temperature, and injected into tobacco leaves.

### 4.7. RNA Extraction and RT-qPCR Analysis

The total RNA was extracted from 100 mg of frozen plant tissue using an RNAprep pure Plant Kit (Tiangen, Beijing, China). The cDNA was synthesized using a FastQuant RT Kit with gDNase (Tiangen, China). Each 20 μL reaction mixture was prepared using SYBR Premix Ex TaqTMII (Takara, Kusatsu, Japan) on an ABI 7500 (ABI, Los Angeles, CA, USA). The RT-qPCR process was as 95 °C for 2 min and 40 cycles of 95 °C for 15 s and 60 °C for 30 s. The gene expression changes were calculated using the 2^−ΔΔCT^ method [56]. At least three independent experiments were performed with three technical replicates for each reaction. The primers used for RT-qPCR analysis are listed in Table S1, using a housekeeping gene (*Actin* 2) in *Arabidopsis thaliana* as the internal references.

## 5. Conclusions and Future Prospects

Carbon-based nanomaterials have always been a hot area of research in plant applications, including for the construction of gene delivery platforms and the promotion of plant growth [57,58,59]. In our study, we chose a carbon dot and found that it localized well in plant cells and could be efficiently enriched in the cell membrane or nucleus within a few minutes. However, the cell membrane and nucleus are important functional organelles in plants, and the subcellular localization of several functional genes occurs in these two organelles. So, CQDs can be used in the future to achieve the delivery function of bio-regulatory elements by adding groups. Secondly, our comparison of CQDs with DAPI shows that CQDs of a high concentration can replace DAPI as an indicator label for subcellular localization of the nucleus in biological research, even with a low toxicity and high efficiency. Furthermore, it has been shown that the CQDs can be transported through the phloem vessels of *Arabidopsis thaliana*, which can be used in the future for fertilization and water transport. Importantly, we have observed that they can enter the floral organs of *Arabidopsis thaliana*, suggesting that the effect on the reproductive development of the plant provides a plasticity for genetic improvement. Of course, the most important aspect of this study is that through the interaction of CQDs with plants, we have shown, using transcriptomics and methylation sequencing, that CQDs cause methylation of the plant genes, which changes the transcription levels of the genes. This is well demonstrated by the influence of the Argonaute gene family. In particular, in the relationship between microRNAs and Argonaute genes, we have used CQDs to uncover a potential new mechanism, namely that the methylation of microRNA172D may directly regulate the expression of the Argonaute gene family.

Taken together, our results suggest that CQDs can freely enter plant organelles, and the data on gene influence may provide a theoretical basis for further modification of CQDs in the future.

## Data Availability

The data presented in this study are available upon request from the corresponding author.

## Supplementary Materials

The following supporting information can be downloaded at: https://www.mdpi.com/article/10.3390/ijms242115700/s1.
