# Supplementary material for: Unlocking the Potential of Carbon Quantum Dots for Cell Imaging, Intracellular Localization, and Gene Expression Control in Arabidopsis thaliana (L.) Heynh
Source: Int J Mol Sci. 2023 Oct 28;24(21):15700. doi: 10.3390/ijms242115700 (PMC10648342; doi:10.3390/ijms242115700)
Supplement: Supplementary file 1 [file ijms-24-15700-s001.zip › ijms-2659795-supplementary.pdf]

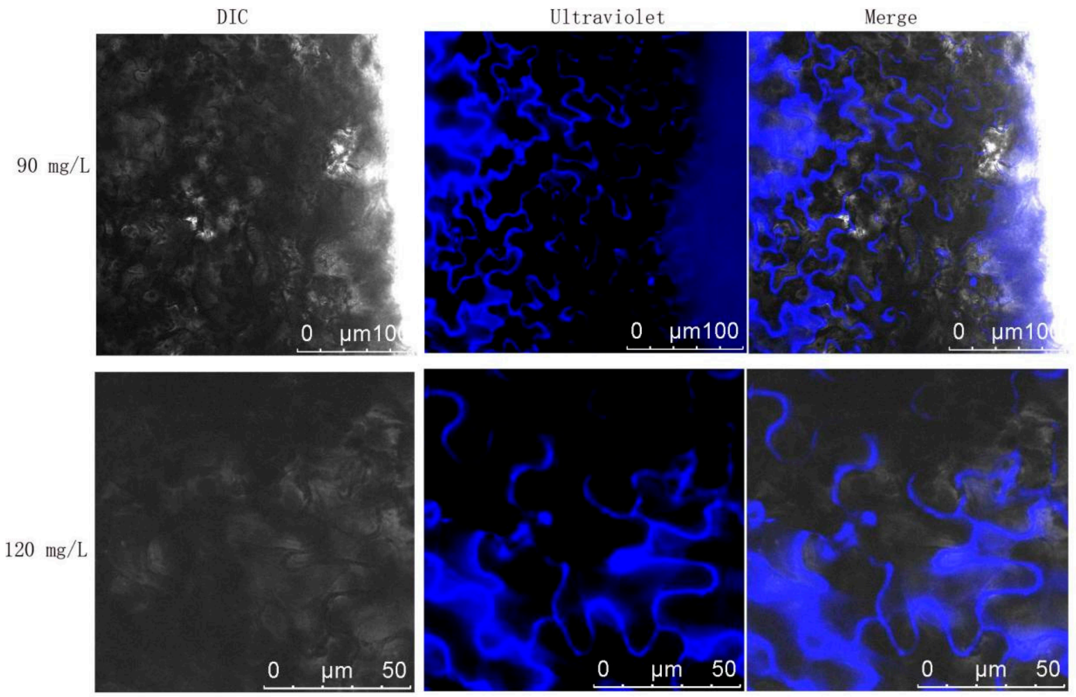

Figure S1. Demonstration of the effect of different concentrations of CQDs on *Arabidopsis* leaves.

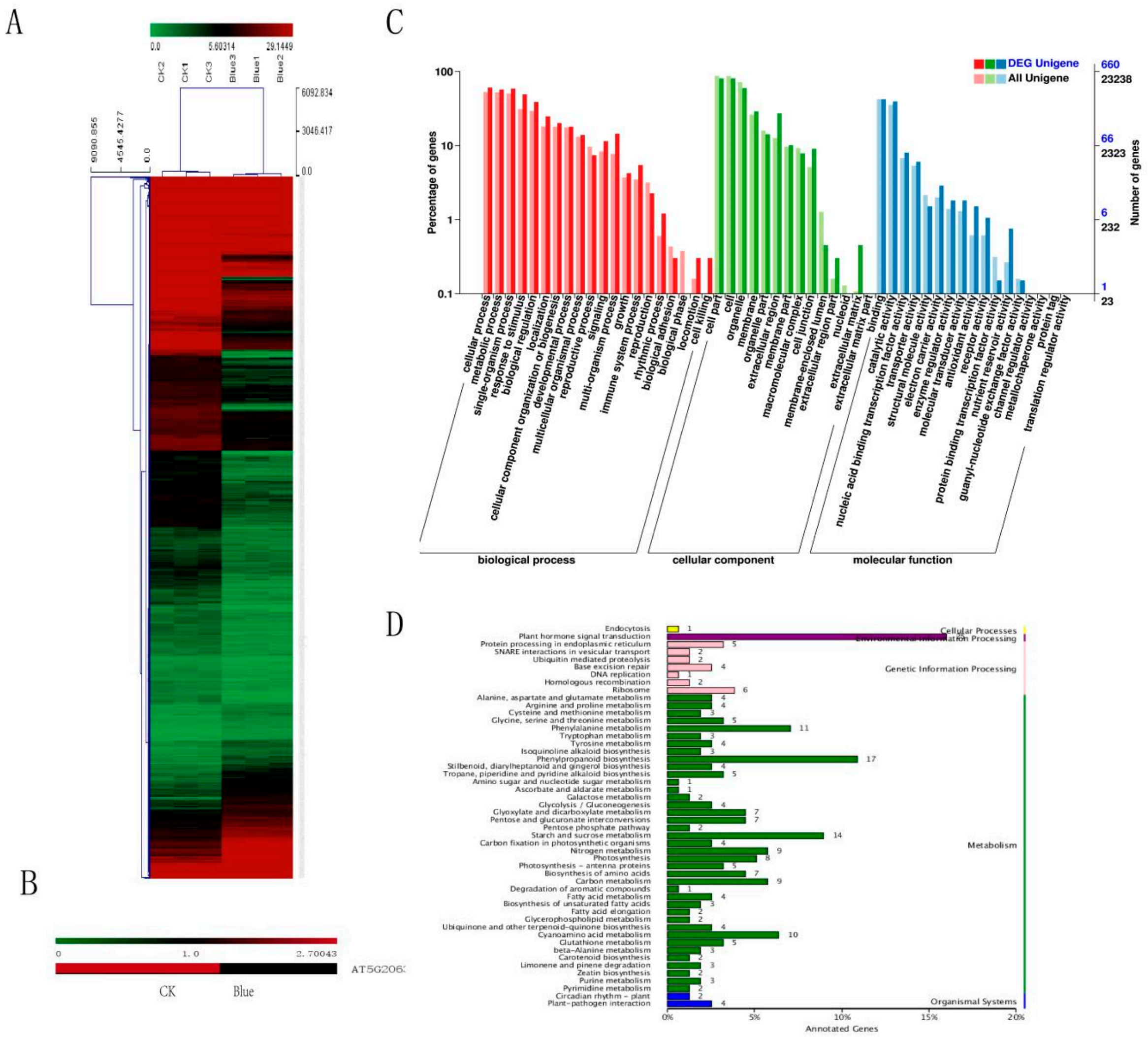

Figure S2. Transcriptome profiling of CK and CQDs-treated *Arabidopsis thaliana*. (A) Heat map indicates the difference in gene expression between the control (CK) and CQDs (Blue) groups. Red and green colors indicate high and low expression levels, respectively. (B) Heat map indicates the difference in AGO3 gene expression between the CK and CQDs. Red and green colors indicate high and low expression levels, respectively. (C) GO has three ontologies: biological processes, cellular components and molecular functions. X axis represents GO term, Y axis represents number of DEG. (D) The pathway classification of DEGs results. X axis represents percentage number of DEG. Y axis represents functional classification of KEGG.

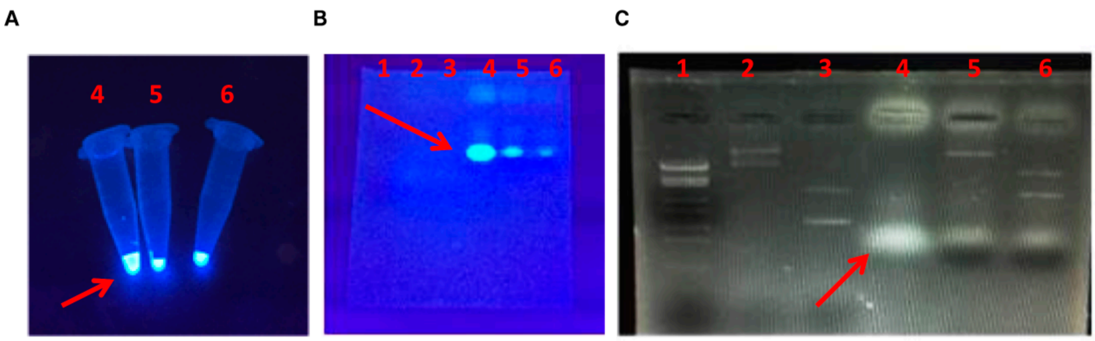

Figure S3. Carbon dot charge analysis by electrophoresis on agarose. (A) Luminescent phenotype of carbon dot observed under UV at three concentrations of 100 mg/mL, 80 mg/mL and 10 mg/mL. (B) The observations under UV irradiation of DNA and Cabon dot after electrophoresis on agarose. Line 1 refer to Marker (λ-EcoT14 I); line 2 refer to circular DNA; line 3 refer to liner DNA; line 4 refer to carbon dot of 100 mg/mL; line 5 refer to carbon dot of 80 mg/mL; line 6 refer to carbon dot of 10 mg/mL. (C)The observations of DNA line. Line 1 refer to Marker (λ-EcoT14 I); line 2 refer to circular DNA; line 3 refer to liner DNA; line 4 refer to carbon dot of 100 mg/mL; line 5 refer to carbon dot of 80 mg/mL; line 6 refer to carbon dot of 10 mg/mL.

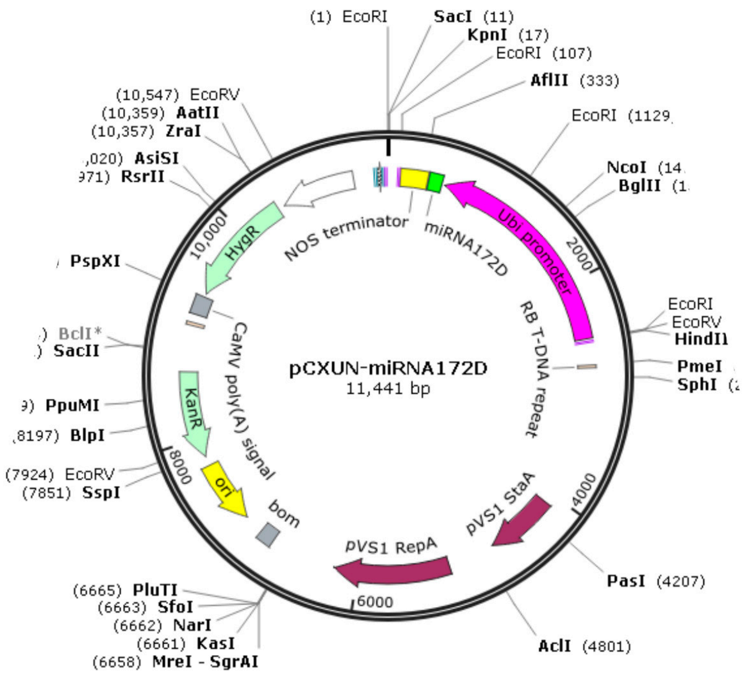

Figure S4. miRNA172D overexpression backbone vector which driven by ubiquitin promoter.

Table S1. Primer sequences used in this study

**Experiment**		**Primer name**	**Sequence (5′–3′)**
PCR amplification	the precursor of microRNA172D (AT3G55512.1)	miF2	AGTCATTGTTTGCTATTGCAACATC
		miR2	AGCCATTTAATTGCCGCTGCAGCAT
qRT-PCR	Internal reference gene (Actin 2)	NactF1	CATGCCATCCTCCGTCTTG
		NactR1	GCTCTGCTGTTGTGGTGAAC
	AGO1 gene (AT1G48410)	A1F	CTGAAGGAGGTGAAGGCTCTGG
		A1R	CTGTTGTTGCTGTTGTGGTGGTT
	AGO2 gene (AT1G31280)	A2F	GTCGTAAGCCACAGGTTCCGTCTG
		A2R	CACAGCCACAACTCCGCCTCTATC
	AGO3 gene (AT1G31290)	A3F2	GGTCGTGGTCGTGGCTTTGTTC
		A3R2	ACTTCCTGTGGCGGAGGTTGTT
	AGO4 gene (AT2G27040)	A4F	GCCGATCTGCTATGCTCACTTGG
		A4R	CATGGAGTTGGCGACGTTGTCTT
	AGO5 gene (AT2G27880.1)	A5F2	GCCTCAACCTGCTATTCCGTTCAT
		A5R2	TTGGCAACACTGAGAGACAATCCC
	AGO6 gene (AT2G32940.1)	A6F	CCTTCAGTAGCAGCGGTTGTTGGT
		A6R	TGCCCTGCTTGTCCGATAGAACTC
	AGO7 gene (AT1G69440)	A7F	TTCCTCCTCCTCCTCCTCCTCATCT
		A7R	CTGTTGGTGTTGCTGCTTTCTCTCC
	AGO8 gene (AT5G21030.1)	A8F	ATGTCTACCAGCGGAGCACAACT
		A8R	GTTGGTGTTCAGCTTCGGCATGG
	AGO9 gene (AT5G21150)	A9F	GCCACAGACGACCTCCAAGAACTT
		A9R	GGACTGCTCCAGGTGTGGTGATTC
	AGO10 gene (AT5G43810)	A10F	GCGGTACTGTTAGTGGCGGTAT
		A10R	CGGTGGCTGATAATTCGGTTCAAG
	CRK gene (AT4G04500)	CRF	TTGTGTGGAAGAGGTGGATTGAAGG
		CRR	GGCATTGTGATAGTAGCGTGTCTCT
	HAK gene (AT4G13420)	HAF	CAGGCTAACGATAACGGTGAAGGAG
		HAR	GCTGTGTGGTTGGAAGTTCAAGTGT
	LEC gene (AT3G24900)	LECF	TCCTTAGTTCCTCAGCGACCATTG
		LECR	CGATAAGTTGAGTGCGATCAGTGC
	PR gene (AT1G15010)	PRF	CGTCATCGACACTCAGGTTCCATC
		PRR	ATGTTCTCGTCCACCGTTCTTCCT
	PAP gene (AT2G01880)	PAPF	ACGGTGTGACTCAAGAGCTTGTAGA
		PAPR	ATGCCTTTGATCCTCCTCCACTTGT
	RLP gene (AT3G24900)	RLF	TCCTTAGTTCCTCAGCGACCATTG
		RLR	CGATAAGTTGAGTGCGATCAGTGC
	SK gene (AT1G66600)	SKF	GCTAACATCCTCAAGACGGTCCTC
		SKR	AAGACTCATGGTGTGGTTGACGATT
PCR amplification		a3F1	ATGGATCGAGGTGGTTACCGAGGAG

	Fusion gene of AGO3 and GFP	a3R1	TCCTCGCCCTTGCTCACCATGACAAAGAACATAAAGTTCTCGATG
		a3F2	AGAACTTTATGTTCTTTGTCATGGTGAGCAAGGGCGAGGAGCTGT
		a3R2	TTACTTGTACAGCTCGTCCATGCCG
